# High risk of adverse birth outcomes among adolescents living with HIV in Botswana compared to adult women living with HIV and adolescents without HIV

**DOI:** 10.1186/s12884-022-04687-y

**Published:** 2022-04-30

**Authors:** Maya Jackson-Gibson, Rebecca Zash, Aamirah Mussa, Ellen C. Caniglia, Modiegi Diseko, Gloria Mayondi, Judith Mabuta, Chelsea Morroni, Mompati Mmalane, Shahin Lockman, Joseph Makhema, Roger L. Shapiro

**Affiliations:** 1 Feinberg School of Medicine Northwestern University, Chicago, Illinois USA; 2 Beth Israel Deaconess Medical Center, Boston, Massachusetts USA; 3 Botswana-Harvard Partnership AIDS Institute, Gaborone, Botswana; 4 University of Pennsylvania Perelman School of Medicine, Philadelphia, Pennsylvania USA; 5 MRC Centre for Reproductive Health, University of Edinburgh, Edinburgh, Scotland; 6 Brigham and Women’s Hospital, Boston, Massachusetts USA; 7 Harvard T. H Chan School of Public Health, Boston, Massachusetts USA

**Keywords:** Adolescent pregnancy, Adverse pregnancy outcomes, HIV, Botswana

## Abstract

**Background:**

Adolescent girls are three times more likely to be living with HIV than boys of the same age. Prior studies have found associations between adolescent pregnancies and increased maternal morbidity and infant mortality, but few studies have assessed the impact of HIV infection on maternal and infant outcomes in adolescents.

**Methods:**

The Tsepamo Study abstracts maternal and infant data from obstetric records in government maternity wards in Botswana. We assessed maternal complications and adverse birth outcomes for all singleton pregnancies from August 2014 to August 2020 at eighteen Tsepamo sites among adolescents (defined as 10–19 years of age) and adults (defined as 20–35 years of age), by HIV status. Univariate and multivariate logistic regression using a complete case analysis method were used to evaluate differences in outcomes.

**Results:**

This analysis included 142,258 singleton births, 21,133 (14.9%) to adolescents and 121,125 (85.1%) to adults. The proportion of adults living with HIV (*N* = 22,114, 22.5%) was higher than adolescents (*N* = 1593, 7.6%). The proportion of most adverse birth outcomes was higher in adolescents. Among adolescents, those with HIV had increased likelihoods of anemia (aOR = 1.89, 95%CI 1.66, 2.15) and cesarean sections (aOR = 1.49, 95%CI 1.3,1.72), and infants with preterm birth (aOR = 1.15, 95%CI 1.0, 1.32), very preterm birth (aOR = 1.35, 95%CI 1.0,1.8), small for gestational age (aOR = 1.37, 95%CI 1.20,1.58), and very small for gestational age (aOR = 1.46, 95%CI 1.20, 1.79).

**Conclusions:**

Adolescent pregnancy and adolescent HIV infection remain high in Botswana. Adolescents have higher risk of adverse maternal and infant birth outcomes than adults, with the worst outcomes among adolescents living with HIV. Linking HIV prevention and family planning strategies for this age group may help minimize the number of infants with poor birth outcomes among this already vulnerable population**.**

## Background

In low-resourced countries, an estimated 2.5 million births occur in girls under age 16 each year, with the highest rates found in Sub-Saharan Africa [1]. Adolescent pregnancy is associated with increased maternal morbidity from post-partum hemorrhage, puerperal endometritis, operative vaginal delivery, episiotomy, and pre-eclamptic/eclamptic events. There is also an increased risk of adverse birth outcomes including preterm delivery and small for gestational age infants with adolescent pregnancy [2–12]. Studies looking to better understand the reasons for these adverse outcomes are conflicting. Some suggest that social determinants of health such as early initiation of smoking, alcohol and substance abuse, low academic interest, single-parent families, poverty, and inadequate prenatal care put adolescents at increased risk [13–16]. Other studies hypothesize biological immaturity to be the underlying cause for this disparity [9, 13, 14, 16–20].

Poor outcomes in adolescent pregnancy may be exacerbated by the HIV epidemic. Studies have shown strong associations between maternal HIV infection in adults with infant mortality and adverse birth outcomes, particularly in low-resourced settings [21–23]. Although these studies have highlighted risks associated with vertical transmission and the potential immunologic impact on HIV-exposed uninfected children, few studies have looked at adverse birth outcomes in the adolescent age group [24]. In Botswana, a country with a high HIV prevalence and high adolescent birth rate (52.3 per 1000), three in every ten new HIV infections occur among adolescents between 15 to 24 years of age. Adolescent girls between 10 to19 years of age are three times more likely to be infected than boys of the same age [25, 26]**.** Using data from a large birth surveillance study in Botswana (the Tsepamo Study), we compared birth outcomes between adolescent girls and adult women by HIV status to better understand the relationship between adolescent HIV infection and adverse pregnancy outcomes.

## Methods

### Study population

Data for this analysis were collected from the ongoing Tsepamo Study, which has performed surveillance of pregnancy and birth outcomes at public hospital delivery sites in Botswana since 2014 [27]. The current analysis includes eighteen study sites that have been operational throughout the entire study period, representing approximately 70% of all births in the country [28]. These maternity wards are located in urban areas (the Princess Marina Hospital in Gaborone, Nyangabgwe Referral Hospital in Francistown) and rural areas (Letsholathebe II Memorial Hospital in Maun, Sekgoma Memorial Hospital in Serowe, the Selebi-Phikwe Hospital, the Mahalapye Hospital, Scottish Livingstone Hospital in Molepolole, and the Ghanzi Primary Hospital). This analysis includes all in-hospital deliveries between August 2014 and August 2020, to women aged 10 to 35 years with singleton births. The upper limit for this age cut off was intended to limit our comparison to younger adults at a standard risk for adverse birth outcomes. Adolescents and adults who delivered twins or triplets were excluded because of the known increased risk of birth complications with multiple gestations [29, 30]. Data were not collected on deliveries that occurred prior to arriving to the hospital, infants transferred from an outside hospital after delivery, or deliveries before 24 weeks gestational age (classified as miscarriage in Botswana).

### Data abstraction

Data were abstracted from the maternal obstetric records at the time of discharge as previously described [28, 31]. For this analysis, abstracted data included hemoglobin levels, initial pregnancy weights, blood pressure measurements, gestational age (GA) at time of delivery, mode of delivery, and infant outcomes. Additional data included demographics, maternal diagnoses during pregnancy, medications and antibiotics prescribed during pregnancy, self-reported alcohol use and smoking, outpatient antenatal care visit dates, and HIV status. For adolescents and women living with HIV, the date of HIV diagnosis and antiretroviral treatment (ART) history (including start date, regimen and any switch or discontinuation during pregnancy) were also recorded. Diagnoses were recorded as per documentation of the treating physician or midwife, except for maternal anemia which was defined as hemoglobin < 10 g/dL and maternal hypertension which was defined as systolic blood pressure ≥ 140 or diastolic blood pressure ≥ 90 in pregnancy. The diagnosis of underweight was defined as initial pregnancy weight < 50 kg [32]. The estimated GA included in the obstetric record was used for analyses and if missing, gestational age was calculated using last menstrual period (LMP). Antenatal clinic nurses estimated GA by date of LMP in most cases but used fundal measurements or ultrasound if LMP was unknown. Infant data were abstracted from the medical record and included sex, birth weight, delivery complications, and admission to the neonatal unit [28, 31].

### Exposures and outcomes

We defined participants 10 to 19 years of age as adolescents and 20 to 35 years of age as adults. The primary outcomes consisted of 1) maternal complications during pregnancy, labor and delivery and 2) adverse birth outcomes. Maternal complications included anemia, diagnosis of syphilis infection, hypertensive disease of pregnancy, and cesarean section. We defined hypertensive disease of pregnancy to include pregnancy induced hypertension and severe hypertension. We acknowledged that there may be unmeasured confounders in the relationships between adolescents and the particular outcomes of interest. Adverse birth outcomes included stillbirth, small for gestational age (SGA), very small for gestational age (VSGA), neonatal death, preterm delivery (PTD), and very preterm delivery (VPTD). Stillbirth was defined as a summed APGAR score of 0. Based on the Intergrowth-21 norms (defined for 24–42 weeks gestational age), infants were categorized as SGA if they weighed less than the tenth percentile birth weight by gestational age, and VSGA if they weighed less than the third percentile birth weight by gestational age [33]. Neonatal death was defined as infant death prior to leaving the hospital within 28 days of delivery. Preterm delivery was defined as deliveries at less than 37 weeks gestational age. Very preterm delivery was defined as deliveries less than 32 weeks gestational age.

### Statistical analysis

The proportions of all outcomes were individually assessed among adolescents and adults overall, then subsequently stratified by HIV status. Chi-square and Fischer exact tests were conducted to compare background information between groups and *p*-values were generated using two-sided tests with an α = 0.01 significance level. Univariate and multivariate logistic regression analyses were performed to evaluate differences in outcomes by age in the overall population and stratified by maternal HIV status. Variables were pre-selected for the logistic regression models based on prior Tsepamo analyses and existing literature and also included all univariate associations *p* < .05. These included Botswana citizenship (yes, no), education (lower education defined as primary school or no schooling, higher education defined as secondary or tertiary schooling), smoking and alcohol use during pregnancy (yes, no), location (urban, rural) to better understand if socioeconomic factors influenced maternal complications and adverse birth outcomes. Additional co-variates included initial pregnancy weight (< 50 kg, 50–80 kg, ≥80 kg, unknown) and HIV status (yes, no) [27, 28, 34–37]. For most of these analyses, adolescents living with HIV are the reference group, aside from the analyses comparing adolescents living without HIV and adults living without HIV. In this model, adolescents living without HIV are the reference group. Statistical analyses were performed using STATA/IC 16.1 software *(STATACorp, College Station, TX)*.

### Ethics approval

Institutional approval for this study was granted by the Health Research and Development Committee in Botswana and by the Office of Regulatory Affairs and Research Compliance at the Harvard T. H. Chan School of Public Health in Boston, Massachusetts.

## Results

**Fig. 1:**
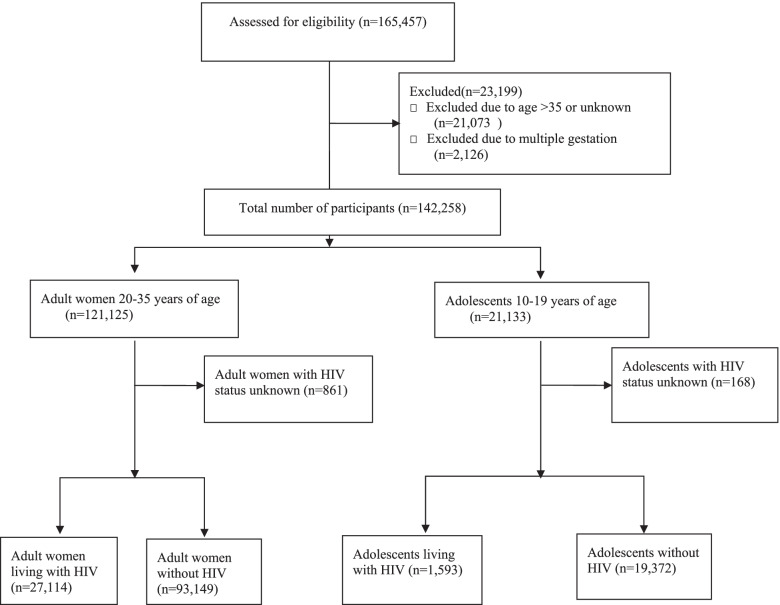
Flow diagram of adolescents and adults living with and without HIV

Between August 2014 and August 2020, the Tsepamo Study recorded 165,457 births and we included 142,258 (86.0%) that met inclusion criteria in this analysis (Fig. 1). Of these, 21,133 (14.9%) were to adolescents (10 to 19 years), with a median age of 18 years, and 121,125 (85.1%) were to adult women (20 to 35 years), with a median age of 26 years. The characteristics of the study population are shown in Table 1. Adolescents were more likely to be unmarried (99.1% vs. 90.4%, *p* < .01), nulliparous (91.0% vs. 35.3%, *p* < .01) and weigh < 50 kg (21.7% vs 12.9%, *p* < .01), while adult women were more likely to have existing hypertension (2.3% vs 0.3%, *p* < .01). Among adolescents, 1593 (7.6%) were living with HIV, compared with 27,114 (22.6%) adult women (*p* < .01). Most adolescents were diagnosed during pregnancy in comparison to adult women (52.6% vs 27.8% *p* < .01). A majority of adolescents and adult women living with HIV were initiated on or continued on ARV therapy during their pregnancies. Adolescents living with HIV were more likely to have a lower education level (11.6% vs 7.3%, *p* < .01) and more likely to be multiparous (14.3% vs 8.5%, *p* < .01) than adolescents living without HIV (Table 1).

**Table 1:** Baseline characteristics of adolescents (10–19 years) and adults (20–35 years), by HIV status

Demographic and Clinical Characteristics	Adolescents living with HIV	Adolescents without HIV	Adults living with HIV	Adults without HIV
	*N* = 1593	*N* = 19,372	*N* = 27,114	*N* = 93,150
Median maternal age, years	18	18	29	25
Community				
Rural	964 (60.5%)	12,650 (65.3%)	15,277 (56.3%)	54,118 (58.1%)
Urban	629 (39.5%)	6721 (34.7%)	11,837 (43.7%)	39,031 (41.9%)
Nationality				
Non-Motswana	23 (1.5%)	294 (1.5%)	763 (2.8%)	3176 (3.4%)
Motswana	1564 (98.6%)	19,008 (98.5%)	26,270 (97.2%)	89,738 (96.6%)
Level of Education				
Higher education	1376 (88.4%)	17, 532 (92.4%)	23,822 (90.2%)	87,091 (95.55%)
Lower education	181 (11.6%)	1448 (7.6%)	2596 (9.8%)	4053 (4.5%)
Marital Status				
Unmarried	1524 (99.5%)	18,573 (99.1%)	24,580 (93.6%)	80,853 (89.5%)
Married	7 (0.5%)	177 (0.9%)	1686 (6.4%)	9479 (10.5%)
Parity				
Nulliparous	1357 (85.7%)	17,666 (91.6%)	4850 (17.9%)	37,568 (40.4%)
Multiparous	227 (14.3%)	1630 (8.5%)	21,798 (80.7%)	54,842 (59.0%)
Grand-multiparous	–	–	381 (1.4%)	487 (0.5%)
Non-Booker				
No	1537 (97.0%)	18,795 (97.5%)	25,894 (96.3%)	90,389 (97.7%)
Yes	47 (3.0%)	474 (2.5%)	1009 (3.8%)	2140 (2.3%)
Earliest Pregnancy Weight				
Underweight	330 (22.1%)	3935 (21.6%)	3165 (12.6%)	11,447 (13.0%)
Normal Weight	1149 (76.9%)	14,072 (77.4%)	20,303 (80.7%)	70,024 (79.5%)
Overweight	15 (1.0%)	178 (1.0%)	1680 (6.7%)	6621 (7.5%)
Alcohol Use and Smoking				
No	1417 (98.6%)	17,631 (99.0%)	23,993 (98.3%)	84,759 (98.9%)
Yes	20 (1.4%)	169 (1.0%)	406 (1.7%)	950 (1.1%)
Existing Hypertensive Disease				
No	1589 (99.8%)	19,315 (99.7%)	26,338 (97.1%)	91,098 (97.8%)
Yes	4 (0.3%)	57 (0.3%)	776 (2.9%)	2052 (2.2%)
HIV Characteristics				
HIV Diagnosis				
Before this pregnancy	723 (45.9%)		19, 209 (71.6%)	
During this pregnancy	830 (52.6%)		7452 (27.8%)	
At delivery	24 (1.5%)		177 (0.7%)	
ART regimen initiated or continued during this pregnancy				
No	138 (8.7%)		1688 (6.2%)	
Yes	1454 (91.3%)		25,423 (93.8%)	
ART use at time of conception				
No	860 (60.0%)		9245 (40.0%)	
Yes	572 (40.0%)		15,766 (60.0%)	

### Adverse maternal and infant outcomes: adolescents vs. adults, by maternal HIV status

**Fig. 2:**
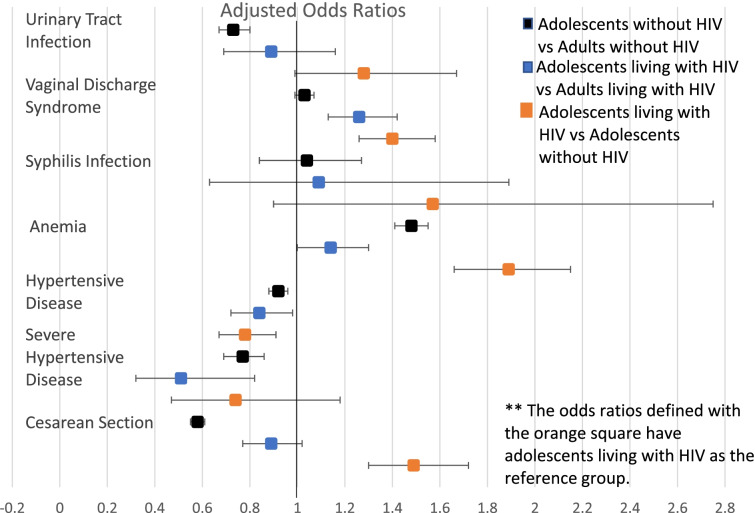
Adjusted Odds Ratios for Maternal Complications

**Fig. 3:**
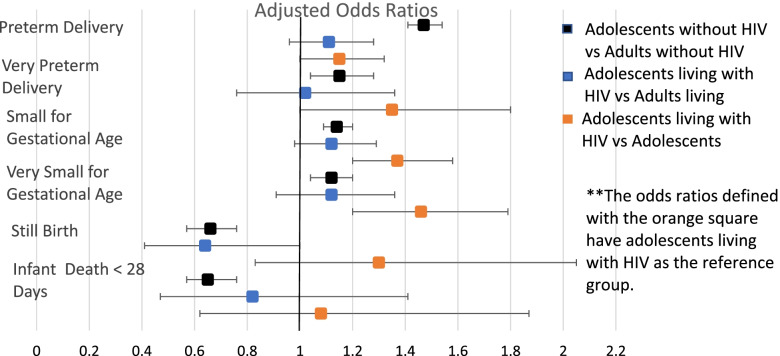
Adjusted Odds Ratios for Adverse Birth Outcomes

Table 2 shows the prevalence of maternal complications and adverse birth outcomes, stratified by age group and HIV status. Figures 2 and 3 show adjusted associations of age group and HIV status with maternal events (Fig. 2) and adverse birth outcomes (Fig. 3). Adjustment for covariates only modestly changed the odds ratios but did not affect the significance of any of the co-variates. Overall, adolescents were more likely than adults to have anemia during pregnancy, PTD, VPTD, SGA infants, and VSGA infants. Adolescents without HIV were more likely to have anemia during pregnancy (aOR = 1.48, 95%CI 1.41, 1.55), but were less likely than adult women living without HIV to be diagnosed with hypertension and severe hypertension (aOR = .92 95%CI .88, .96 and aOR = 0.77, 95%CI .69, .86 respectively). Additionally, these adolescents without HIV were more likely to have PTD (aOR = 1.47, 95%CI 1.41, 1.54), VPTD (aOR =1.15, 95%CI 1.02,1.28), SGA infants (aOR = 1.14, 95%CI 1.09,1.20), and VSGA infants (aOR = 1.12, 95%CI 1.04,1.20). Adolescents living without HIV were less likely to have cesarean sections at time of delivery (aOR = .58, 95%CI .55, .61) compared to adults without HIV. Among everyone living with HIV, adolescents were more likely to be anemic (aOR = 1.14, 95%CI 1.0,1.30) during pregnancy. Adolescents living with HIV were also more likely to have PTD (aOR = 1.02, 95%CI .76, 1.36), SGA infants (aOR = 1.12, 95%CI .98, 1.29), and VSGA infants (aOR = 1.12, 95% CI .91, 1.36). When controlled for ART use at time of conception, we found no difference in maternal complications and adverse birth outcomes between adolescents living with HIV and adults living with HIV.

**Table 2:** Maternal complications and adverse birth outcomes, by HIV status

	Overall		Living with HIV		Without HIV	
	Adolescents ***N*** = 21,133^a^	Adults ***N*** = 121,125^a^	Adolescents ***N*** = 1593^a^	Adults ***N*** = 27,114^a^	Adolescents ***N*** = 19,372^a^	Adults ***N*** = 93,150^a^
Maternal Complications						
Urinary Tract Infection	3.6%	4.7%	4.3%	4.8%	3.5%	4.7%
Vaginal Discharge Syndrome or STD	31.6%	31.4%	38.0%	32.8%	31.2%	31.2%
Syphilis Infection	0.8%	0.9%	1.3%	1.2%	0.8%	0.8%
Anemia	18.5%	15.5%	28.8%	25.9%	17.7%	12.6%
Hypertensive disease	17.2%	19.2%	14.5%	17.2%	17.4%	19.8%
Severe Hypertensive Disease	14.3%	18.5%	11.3%	19.8%	14.5%	18.2%
Cesarean Section	14.1%	22.1%	19.4%	21.8%	13.7%	22.2%
Adverse Birth Outcomes						
Preterm birth	19.5%	15.4%	21.9%	20.1%	19.0%	13.8%
Very preterm birth	3.8%	3.6%	4.6%	4.7%	3.5%	
Small for gestational age	17.5%	15.3%	22.3%	18.6%	17.1%	14.3%
Very small for gestational age	6.3%	5.7%	8.8%	7.4%	6.1%	5.2%
Stillbirth at delivery	1.6%	1.5%	1.8%	2.7%	1.5%	2.0%
Infant death < 28 Days	1.2%	1.4%	1.3%	1.6%	1.1%	1.3%

^a^Available data for each covariate differed: for urinary tract infection, *N* = 1494 adolescents, 25,159 adults; for vaginal discharge syndrome or STD, *N* = 1494 adolescents, 25,159 adults; for syphilis infection, *N* = 1186 adolescents, 20,927 adults; for anemia, *N* = 1359 adolescents, 23,815 adults; for hypertensive disease, *N* = 1486 adolescents, 25,076 adults; for severe hypertensive disease, *N* = 216 adolescents, 4311 adults; for cesarean section, *N* = 1494 adolescents, 25,144 adults; for preterm birth, *N* = 1446 adolescents, 24,673 adults; for very preterm birth, *N* = 1446 adolescents, 24,673 adults; for small for gestational age, *N* = 1427 adolescents, 24,471 adults; for very small for gestational age, *N* = 1427 adolescents, 24,471 adults; for still birth at delivery, *N* = 1493 adolescents, 25,158 adults; for infant death < 28 days, *N* = 1468 adolescents, 24,548 adults;

### Adverse maternal and infant outcomes: adolescents living with HIV vs adolescents without HIV

Adolescents living with HIV were more likely to be diagnosed with anemia (aOR = 1.89 CI 1.67, 2.14), more likely to have cesarean sections (aOR = 1.49 CI 1.30,1.72), and less likely to be diagnosed with hypertension during pregnancy (aOR = 0.78 CI 0.67, 0.91), than adolescents without HIV (Fig. 2). Additionally, adolescents living with HIV were more likely to have PTD (aOR = 1.15 CI 1.04,1.28), VPTD (aOR = 1.35 CI 1.0, 1.8), SGA infants (aOR = 1.37 CI 1.20,1.58), and VSGA infants (aOR = 1.46 CI 1.20,1.79).

## Discussion

We evaluated adverse maternal and birth outcomes among adolescents and adult women by HIV status in a large, nationally representative sample in Botswana. Adolescents had a greater likelihood than adults of anemia, and for infants with preterm birth, very preterm birth, small for gestational age, and very small for gestational age. Adolescents living with HIV were at the highest risk for these adverse outcomes. To our knowledge, this is the first study directly comparing maternal and birth outcomes of adolescents living with HIV to adults and adolescents living without HIV.

Consistent with findings from previous studies conducted in both low and high income countries, we found that adolescents have increased maternal complications during pregnancy and adverse birth outcomes when compared to adult women [16, 38, 39]. Multiple prior studies reported that socioeconomic factors are the main drivers for the increased susceptibility of adolescents to adverse outcomes [9, 16, 40, 41]. Our findings do not appear to be related to the socioeconomic factors that we could measure (education level, nationality, location, alcohol and tobacco use), but we cannot say this for certain. It is possible that our available socioeconomic indicators were insufficient to detect subtle differences in our study (or other sources of unmeasured confounding were present). It should be acknowledged that biological immaturity among adolescents may explain some of these differences.

We identified differences in birth outcomes by HIV status that were more pronounced among adolescents than adults. Prior studies have found that infants born to women living with HIV experience worse birth outcomes than those who are uninfected, even when they are on effective antiretroviral medication with viral suppression and high CD4 cell counts [21]. We found that 52.6% of adolescents living with HIV were diagnosed during their pregnancy in comparison to 27.8% of adults. We also discovered that adolescents living with HIV were more likely to have several adverse outcomes when compared to adults living with HIV. While we cannot exclude socioeconomic differences, potential biological explanations include young gynecologic age (defined as conception within 2 years of menarche), altered prostaglandin production, immaturity of the uterine blood supply coupled with altered angiogenesis and low weight gain due to existing HIV infection [9, 42]. Additionally, ART adherence among adolescents may have differed from adults, and has been shown to be lower in prior studies, but we did not have adherence data available [43].

Our data reveal that adolescents living with HIV had even more adverse outcomes than adolescents without HIV, with both groups experiencing even worse outcomes than adults living with or without HIV. The increase of adverse maternal and infant outcomes among adolescents living with HIV, highlights the importance of accessible pregnancy family planning and contraceptive methods, routine HIV screening, and the accessibility of HIV prevention methods among this population. In 2004, Botswana began to perform routine HIV screening of women in the prenatal setting based on the joint recommendation by the United Nations and World Health Organizations [44]. Although routine screening for HIV during antenatal visits is vitally important for the diagnosis and treatment of new HIV infections, a continued effort also needs to be placed on screening prior to pregnancy in places such as schools or at home using HIV self-testing kits. There is also the need to increase access to and promote the acceptability of HIV prevention methods, such as PrEP, to decrease the rate of new infections amongst this high-risk population.

Lower weight is highly associated with poor birth outcomes and it is more prevalent in adolescents. We found that 22.1% of our adolescents living with HIV and 21.6% of adolescents without HIV were underweight. While we adjusted for low weight upon the patient’s initial weight recorded during pregnancy in our analyses, we found that weight was not the only significant confounder in the overall model, so it is difficult to conclude how it impacts birth outcomes. In contrast to other outcomes, our study suggests that adolescents living with HIV were less likely to be diagnosed with hypertension during pregnancy than adolescents without HIV, even after adjusting for early pregnancy weight. Adolescents without HIV are thought to be at higher risk of experiencing pre-eclampsia and eclampsia during pregnancy in comparison to adult women, but we were unable to capture the necessary labs to identify preeclampsia in this study [12, 41].

The strengths of this study include a large sample size, a well-defined population of adolescent and adult women who were pregnant, and the ability to assess multiple maternal complications and adverse birth outcomes. We acknowledge that our study is not without its limitations. Diagnostics were unavailable to distinguish pre-eclampsia from other forms of hypertensive disease of pregnancy, and we were unable to confirm the diagnoses of STDs and UTIs, which could contribute to adverse birth outcomes. Gestational dating was largely dependent on last menstrual period assessed by midwives, which may be less accurate than early ultrasound; however, we would not expect differential bias between exposure groups. Because almost all women living with HIV received antiretroviral treatment, we were unable to distinguish differences in outcomes by the presence or absence of antiretroviral exposure. Lastly, this was an observational study and prone to both measured and unmeasured confounding.

## Conclusions

The combination of adolescent pregnancy and HIV infection in Botswana remains of great concern**.** The resulting adverse maternal and birth outcomes continue to negatively impact this age-group disproportionately, despite available ART. Our findings reinforce the importance of high-risk obstetrical and pediatric support for adolescents with HIV, and support public health measures aimed at decreasing adolescent pregnancy and incident HIV to improve health outcomes in Botswana.

## Data Availability

The datasets used and/or analyzed during the current study are available from the corresponding author on reasonable request.

## Abbreviations

ART: Anti-retroviral therapy
GA: Gestational age
LMP: Last menstrual period
PTD: Preterm delivery
SGA: Small for gestational age
VPTD: Very preterm delivery
VSGA: Very small for gestational age
